# A Three Protein-Coding Gene Prognostic Model Predicts Overall Survival in Bladder Cancer Patients

**DOI:** 10.1155/2020/7272960

**Published:** 2020-10-10

**Authors:** Xiang-hui Ning, Yuan-yuan Qi, Fang-xin Wang, Song-chao Li, Zhan-kui Jia, Jin-jian Yang

**Affiliations:** ^1^Department of Urology, The First Affiliated Hospital of Zhengzhou University, China; ^2^Department of Nephrology, The First Affiliated Hospital, Zhengzhou University, China; ^3^Department of Oral & Maxillofacial Surgery, The First Affiliated Hospital, Zhengzhou University, China

## Abstract

Bladder cancer (BLCA) is the most common urinary tract tumor and is the 11th most malignant cancer worldwide. With the development of in-depth multisystem sequencing, an increasing number of prognostic molecular markers have been identified. In this study, we focused on the role of protein-coding gene methylation in the prognosis of BLCA. We downloaded BLCA clinical and methylation data from The Cancer Genome Atlas (TCGA) database and used this information to identify differentially methylated genes and construct a survival model using lasso regression. We assessed 365 cases, with complete information regarding survival status, survival time longer than 30 days, age, gender, and tumor characteristics (grade, stage, T, M, N), in our study. We identified 353 differentially methylated genes, including 50 hypomethylated genes and 303 hypermethylated genes. After annotation, a total of 227 genes were differentially expressed. Of these, 165 were protein-coding genes. Three genes (zinc finger protein 382 (*ZNF382*), galanin receptor 1 (*GALR1*), and structural maintenance of chromosomes flexible hinge domain containing 1 (*SMCHD1*)) were selected for the final risk model. Patients with higher-risk scores represent poorer survival than patients with lower-risk scores in the training set (HR = 2.37, 95% CI 1.43-3.94, *p* = 0.001), in the testing group (HR = 1.85, 95% CI 1.16-2.94, *p* = 0.01), and in the total cohort (HR = 2.06, 95% CI 1.46-2.90, *p* < 0.001). Further univariate and multivariate analyses using the Cox regression method were conducted in these three groups, respectively. All the results indicated that risk score was an independent risk factor for BLCA. Our study screened the different methylation protein-coding genes in the BLCA tissues and constructed a robust risk model for predicting the outcome of BLCA patients. Moreover, these three genes may function in the mechanism of development and progression of BLCA, which should be fully clarified in the future.

## 1. Introduction

Bladder cancer (BLCA) is the most common urinary tract tumor and the 11th most malignant tumor worldwide. It is estimated that 80,470 new cases and 17,670 new deaths occurred in America in 2019 [1]. Muscle invasive bladder cancer (MIBC) is a more aggressive form of the disease, with a 5-year survival rate of approximately 60% in patients with local disease and less than 5% in patients with distant metastasis [2]. The classical factors that are closely associated with the prognosis of BLCA include tumor clinical stage, tumor grade, carcinoma in situ, and distant metastasis. In the past decade, the study of molecular markers to predict the prognosis of BLCA has expanded and has focused mainly on DNA alterations, such as DNA polymorphisms, mutations, and mRNA and protein expression levels [3]. In recent years, with the development of in-depth multisystem sequencing, an increasing number of prognostic molecular markers have been identified [4]. Molecular biomarkers can effectively improve the accuracy of prognosis prediction and may also highlight tumorigenic characteristics that are helpful for the development of novel therapies, as well as identifying mechanisms underpinning the development and progression of diseases.

Epigenetic alteration can affect the development and progression of tumors. The main epigenetic processes and modifications associated with cancer biology can be divided into three categories: abnormal DNA methylation, chromatin remodeling (including histone modification), and regulation by noncoding RNA [5]. Of these biological processes, DNA methylation is the most studied epigenetic alteration in BLCA. DNA hypermethylation occurs in approximately 50-90% of BLCAs. The hypermethylated genes can be further clustered into tumor suppressor genes, DNA repair genes, cell cycle control genes, and cell invasion protein-coding genes (e.g., RNA-binding fox-1 homolog 1 (*RBFOX1*), telomerase reverse transcriptase (*TERT*), neuronal pentraxin 2 (*NPTX2*), SRY-box transcription factor 11 (*SOX11*), and homeobox A9 (*HOXA9*)). Changes in the methylation status of these genes may promote the development or progression of BLCA. In addition, DNA hypomethylation is also seen in BLCA, but its effect is still unclear [5].

Based on the above evidence, investigations into the prognosis of BLCA have now extended into other forms of epigenetic regulation. For example, miRNA, lncRNA, other noncoding RNAs, and DNA methylation are also used to predict the prognosis of BLCA.

For instance, abnormal expression of lncRNAs such as HOX transcript antisense RNA (*HOTAIR*) and growth arrest specific 5 (*GAS5*) is associated with disease-free survival and disease-specific survival of patients with BLCA [6]. It has also been reported that abnormal DNA methylation of different genes such as aldehyde dehydrogenase 1 family member A3 (*ALDH1A3*), protocadherin 8 (*PCDH8*), Ras association domain family member 1 (*RASSF1*), and RUNX family transcription factor 3 (*RUNX3*) is independently associated with the prognosis of BLCA [7]. Indeed, prognosis-predicting models based on gene methylation have been developed in recent years. Six genes, namely, Rho GDP dissociation inhibitor beta (*ARHGDIB*), long intergenic non-protein-coding RNA 526 (*LINC00526*), isocitrate dehydrogenase (NADP(+)) 2 (*IDH2*), ADP ribosylation factor like GTPase 14 (*ARL14*), glutathione S-transferase mu 2 (*GSTM2*), and leucine-rich adaptor protein 1 (*LURAP1*), have been identified for the development of risk models to predict BLCA prognosis. However, this methylation risk model includes LINC00526, which is a noncoding RNA, and the role of lncRNA gene methylation in cancer has not been well studied [8].

Therefore, our study is focused on the role of protein-coding gene methylation in the prognosis of BLCA. We describe a new risk model including protein-coding gene methylation which may not only improve the methods of predicting BLCA prognosis but can also screen genes with clear biological effects, which will help in identifying the mechanisms involved in BLCA development and progression.

## 2. Materials and Methods

### 2.1. BLCA Clinical and Methylation Data Acquisition

BLCA clinical data and methylation data from the HumanMethylation450 BeadChip were downloaded from The Cancer Genome Atlas (TCGA) database according to the publication guidelines (https://portal.gdc.cancer.gov/). Patients whose data fulfilled the enrollment criteria were included in our study. The enrollment criteria were as follows: survival status, age, gender, and clear information regarding tumor characteristics (tumor grade, clinical stage, and Tumor/Node/Metastasis (TNM) stage), with a survival time of longer than one month. All the datasets used throughout this study were publicly available (https://portal.gdc.cancer.gov/).

### 2.2. Differential Methylation Analysis and Construction of Survival Model

The Wilcox test was used to compare the differential methylation status of genes in tumor and normal tissues, and genes with *p* < 0.05 and log fold change > 1 were considered to be differentially methylated. These genes were annotated using an annotation program obtained from GENCODE (https://www.gencodegenes.org/). Total tumor samples were randomly divided into training and testing sets using the set seed method. Then, lasso (least absolute shrinkage and selection operator) regression was used to identify the eligible protein-coding genes for construction of a BLCA prognostic signature using the screened methylation protein-coding genes, which generated corresponding coefficients for each sample [9]. Lasso regression analysis was conducted using the “glmnet” package in the R software suite [10]. And the optimal value of the penalty parameter *λ* was determined by tenfold cross-validations. The best *λ* value (lambda.min) was used to identify the proper genes and their coefficients to construct the gene risk model. In the training set, the risk score for each patient was calculated and the data were divided into high-risk and low-risk groups according to the median risk score. In the testing set and the total set, the same procedure was performed to validate the model.

### 2.3. Statistical Analyses

Overall survival was assessed using the Kaplan-Meier method and the log-rank test. The hazard ratio (HR) was calculated using a Cox regression model, and the result was provided as HR value with a 95% confidence interval. Variables with *p* values of less than 0.05 were subjected to univariate Cox regression, in a multivariate Cox regression test using a backward conditional approach which eliminated the variables with a *p* value of >0.05.

Statistical tests were conducted using R software and SPSS 19.0 software (SPSS, Inc., Chicago, IL, USA). A *p* value of <0.05 was considered to be statistically significant.

## 3. Results

### 3.1. Characteristics of BLCA Clinical Data

Four hundred and twelve cases of clinical data from patients with BLCA were downloaded from TGCA database. Of these, 365 cases fulfilled the enrollment criteria and were included in our study. The demographic and clinical characteristics of these patients with BLCA are shown in Table 1. The median age of BLCA patients was 69 years (34-89 years). Male patients were in the majority, accounting for 74.0% (270/365) of the cases. There were 348 cases with high-grade tumors (95.3%) and 38.9% (142/365) died during follow-up. The total samples were randomly divided into the discovery group and the verification group, and no significant statistical difference was observed between the clinical data (Table 1).

### 3.2. BLCA Differentially Methylated Genes

The HumanMethylation450 BeadChip data of 440 cases in the BLCA dataset, including 21 normal and 419 cancer samples, were downloaded. Differential methylation analysis identified 353 differentially methylated genes, which included 50 hypomethylated and 303 hypermethylated genes (Supplementary Figure 1). We annotated a total of 227 differentially expressed genes, of which 165 genes were protein-coding genes.

### 3.3. Three Methylated Protein-Coding Genes Are Related to Overall Survival of Patients with BLCA

**Figure 1:**
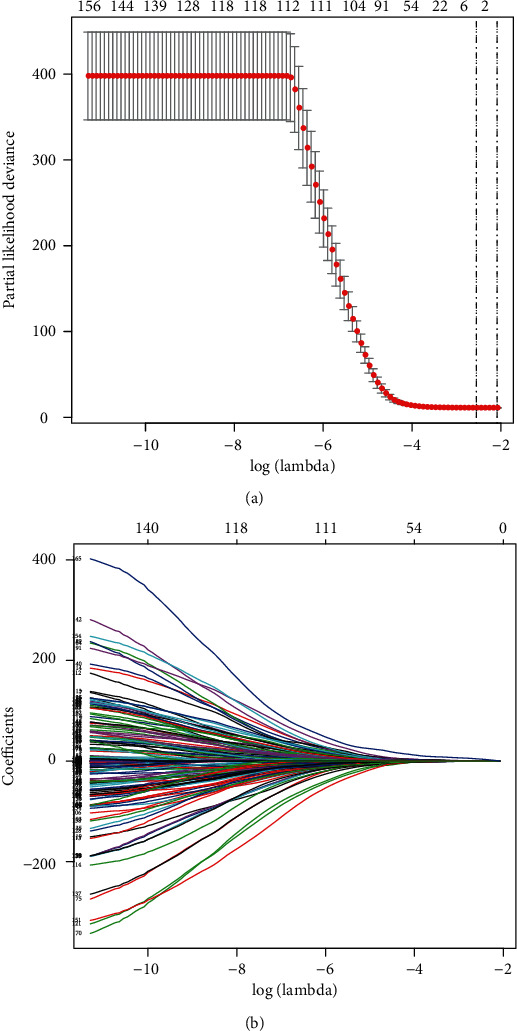
Screening the prognostic genes in BLCA patients using the lasso regression method. (a) The plot displays the cross-validation error according to the log of lambda. (b) The coefficients of each gene in lasso regression.

**Figure 2:**
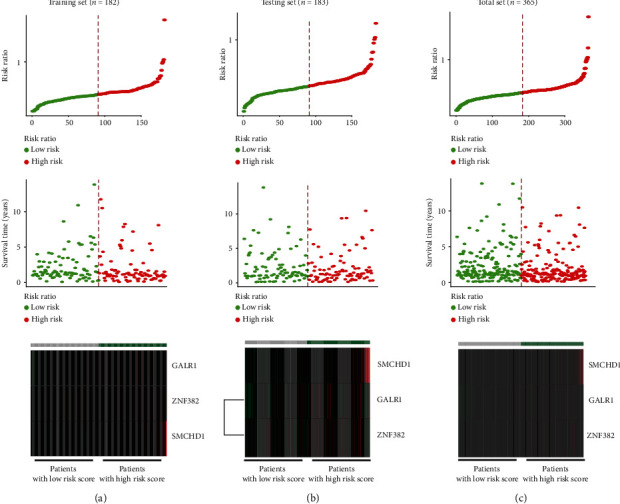
The risk score distribution, survival overview, and heat map of three protein-coding genes in these three groups. The first figure in each group represents the risk score distribution in the two groups. The second figure in each group indicates the overall survival status. The third figure in each group lists the heat map of three genes' methylation conditions. (a) The training set. (b) The testing set. (c) The total set.

**Figure 3:**
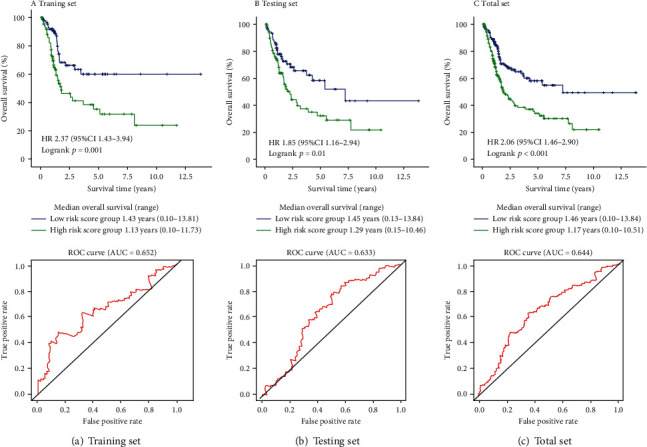
Kaplan-Meier plots and ROC curve for 3-year survival prediction of the training set, testing set, and total set. (a) The training set. (b) The testing set. (c) The total set.

**Figure 4:**
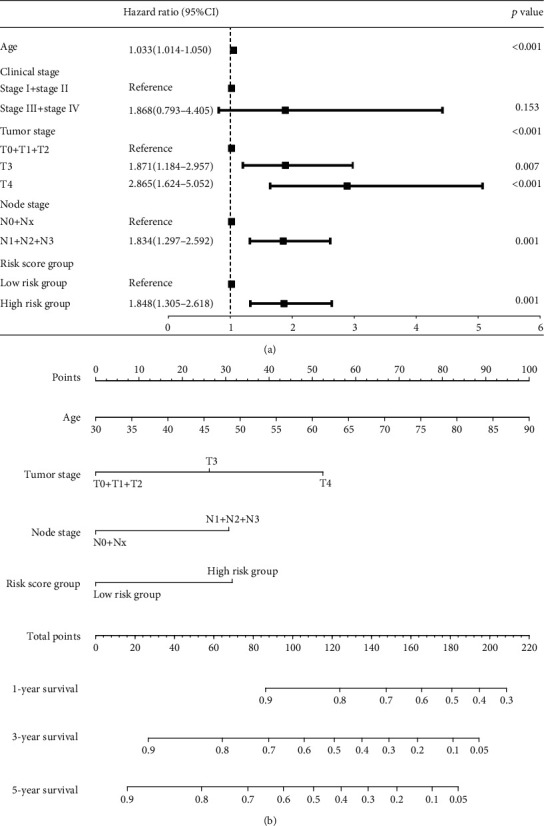
Forest map and a nomogram for overall survival (OS) prediction in BLCA patients derived from the total set. (a) The variables in the multivariate Cox regression results for predicting the overall survival of BLCA patients. (b) The composite nomogram consists of the three protein-coding gene methylation risk score, tumor stage, and age. Each variable can generate a point according to the “Points” line. Add these three points together and get the total points on the “Total Points” line. Then, draw a vertical line from the “Total Points” line to the three lines below which correspond to the predicted 1-year, 3-year, and 5-year OS rates by the nomogram.

Based on the training set, we used a multivariate lasso Cox regression model to establish a prognosis risk model based on the methylation status of the 165 protein-coding genes. Throughout the lasso regression, the minimal *λ* was 0.07802942 and three genes (zinc finger protein 382 (ZNF382), galanin receptor 1 (GALR1), and structural maintenance of chromosomes flexible hinge domain containing 1 (SMCHD1)) were screened for the final risk model (Figure 1). Utilizing the lasso Cox regression model, we also calculated the risk score of each patient based on the methylation values of these three genes, as follows: Risk score = (0.2337∗ZNF382) + (0.4722∗GALR1) + (4.0402∗SMCHD1) (Figure 2). Patients with higher-risk scores presented with poorer survival than patients with lower-risk scores (HR = 2.37, 95% CI 1.43-3.94, *p* = 0.001; Figure 3). To validate these risk models, we conducted analyses in the testing and the total data sets. The validation results showed that patients with higher-risk scores had better survival than patients with a lower-risk score in the testing group (HR = 1.85, 95% CI 1.16-2.94, *p* = 0.01; Figure 3), as well as in the total cohort (HR = 2.06, 95% CI 1.46-2.90, *p* < 0.001; Figure 3). Further univariate and multivariate analyses using the Cox regression method were conducted in all three groups. All the results indicated that risk score was an independent risk factor for BLCA (Table 2). The results developed using the total set were used to make the forest map and the nomogram (Figure 4).

## 4. Discussion

Our study proposes a novel survival model, based on methylation of protein-coding genes, for predicting prognosis of patients with BLCA. Three genes (*ZNF382*, *GALR1*, and *SMCHD1*) were selected for the risk model.

Galanin receptor 1 (*GALR1*) is a protein-coding gene that is widely expressed in the brain, spinal cord, around the small intestine, and heart. Many studies have demonstrated that GALR1 plays a tumor suppressor role in different types of cancers. GALR1 has been intensively studied in head and neck squamous cell carcinoma (HNSCC). These investigations revealed that the *GALR1* promoter was widely hypermethylated in HNSCC cell lines and primary tumor specimens, and its methylation was closely related to reduce expression of GALR1. In addition, the expression of GALR1 could be recovered by treatment with the histone deacetylase inhibitor, trichostatin A, and the methyltransferase inhibitor, 5-azacytodine [11]. The status of *GALR1* methylation may be an important site-specific biomarker for predicting clinical outcomes in HNSCC patients, and assessing methylation of its promoter can play a role in risk stratification for individualized treatment [12, 13]. *GALR1* has also been used in the formation of a risk model to diagnose non-small-cell lung cancer (NSCLC) [14]. Furthermore, DNA methylation of *GALR1* is the most frequent epigenetic change in endometrial cancer, and the detection of *GALR1* methylation in vaginal swabs can accurately identify female endometriosis and malignant changes [15]. Our study also suggests that *GALR1* gene methylation is involved in the prognosis of BLCA, but its role in this cancer is still unknown.

The zinc finger protein 382 (*ZNF382*) gene encodes the KRAB domain zinc finger transcription factor (KZNF). KZNF is widely involved in development and tumorigenesis. It plays a key role in the regulation of various cellular processes, including differentiation, proliferation, and apoptosis. It acts as a tumor suppressor and is often methylated in many cancers. ZNF382 is frequently downregulated by promoter methylation in HBV-associated hepatocellular carcinoma (HCC), and decreased expression of ZNF382 is closely linked to poor survival in patients with early HCC. ZNF382 is an effective tumor suppressor in HCC cells. Functional studies have shown that it inhibits cell proliferation, colony formation, migration, invasion, and tumorigenic potential in nude mice and induces apoptosis [16]. *ZNF382* is also methylated and exhibits reduced expression in gastric cancer (GC) tissues. Pei et al. showed that ZNF382 can reverse the process of epithelial to mesenchymal transition in GC cells through NOTCH signaling, further supporting its role as a tumor suppressor [17]. In esophageal squamous cell carcinoma (ESCC), the expression of ZNF382 was inhibited due to aberrant promoter methylation and *ZNF382* methylation correlated with the level of ESCC differentiation. ZNF382 also inhibited the proliferation and metastasis of ESCC cells by inhibiting the Wnt/beta-catenin signaling pathway. These data suggest that ZNF382 plays a role in inhibiting the formation of ESCCs [18]. *ZNF382* is also methylated in a range of primary tumors (nasopharyngeal, esophageal, colon, stomach, and breast). Ectopic expression of ZNF382 in silenced tumor cells significantly inhibited their cloning and proliferation and induced apoptosis. Cheng et al. showed that ZNF382 can inhibit nuclear factor kappa-B and AP-1 signaling and that it downregulates the expression of several oncogenes [19]. However, the role of ZNF382 in BLCA has not been well established and is worthy of further investigation.

Structural maintenance of chromosomes flexible hinge domain containing 1 (*SMCHD1*) is a protein-coding gene. Mutations in *SMCHD1* have been associated with mastoid microocular syndrome and facial-shoulder-brachial dystrophy 2. The SMCHD1 protein is involved in DNA methylation, and a number of studies have focused on its contribution to X chromosome inactivation. SMCHD1 plays a role in the normal development of the nose, eyes, and other structures of the head and face and appears to be involved in repairing damaged DNA. In response to DNA damage, SMCHD1 is recruited to sites of DNA double-strand breakage, where it promotes repair of the breakage by nonhomologous end joining (NHEJ), while inhibiting repair by homologous recombination [20, 21]. One study suggested that SMCHD1 may be a candidate tumor suppressor gene in prostate cancer [22]. However, there has been comparatively little research into the role of SMCHD1 in different cancer types, so whether it functions as a tumor suppressor or an oncogene is not yet clear. In this study, we found that the *SMCHD1* gene was hypomethylated in BLCA tumors. DNA hypomethylation has been shown to cause the abnormal activation of a few genes. The mechanistic links between the loss of DNA methylation and cancer development, including induction of chromosomal instability, reactivation and transposition of reversed loci, loss of imprinting, and activation of normally silenced genes, are directly related to DNA methylation patterns in mammalian genomes [23]. Future studies should focus on identifying the role of SMCHD1 in BLCA.

In this study, we construct a three-gene model for predicting the prognosis of BLCA using TCGA dataset. However, one limitation of our study is the lack of an independent dataset for validating our results. In addition, our study is an observational study, and the gene model should be verified by prospective studies in the future.

## 5. Conclusion

Our study screened differentially methylated protein-coding genes in BLCA tissues and constructed a robust risk model for predicting the survival of BLCA patients. Moreover, the three genes which form the basis of our risk model may function in the development and progression of BLCA and represent worthwhile avenues for future investigations.

## Figures and Tables

**Table 1:** Bladder cancer (BLCA) patients' demographics and clinical characteristics.

Variable	Training set	Testing set	*p* value	Total set
Sample (*n*)	182	183		365
Age (range)			0.419	
	70 (34-87)	68 (43-89)		69 (34-89)
Gender			0.572	
Male	137 (75.3%)	133 (72.7%)		270 (74.0%)
Female	45 (24.7%)	50 (27.3%)		95 (26.0%)
Grade			0.08	
High grade	170 (93.4%)	178 (97.3%)		348 (95.3%)
Low grade	12 (6.6%)	5 (3.7%)		17 (4.7%)
Clinical stage			0.657	
Stage I	1 (0.5%)	1 (0.5%)		2 (0.5%)
Stage II	54 (29.7%)	45 (24.6%)		99 (27.1%)
Stage III	69 (37.9%)	69 (37.7%)		138 (37.8%)
Stage IV	58 (31.9%)	68 (37.2%)		126 (34.5%)
Tumor stage			0.850	
T0+T1	3 (1.6%)	1 (0.5%)		4 (1.1%)
T2	56 (30.8%)	56 (30.6%)		112 (30.7%)
T3	95 (52.2%)	97 (53.0%)		192 (52.6%)
T4	28 (15.4%)	29 (15.8%)		57 (15.6%)
Metastasis stage			0.171	
M0	91 (50.0%)	82 (44.8%)		173 (47.4%)
M1	6 (3.3%)	2 (1.1%)		8 (2.2%)
Mx	85 (46.7%)	99 (54.1%)		184 (50.4%)
Node stage			0.070	
N0	109 (59.9%)	107 (58.5%)		216 (59.2%)
N1+N2+N3	55 (30.2%)	68 (37.2%)		123 (33.7%)
Nx	18 (9.9%)	8 (4.4%)		26 (7.1%)
Survival status			0.300	
Alive	116 (63.7%)	119 (64.0%)		223 (61.1%)
Died	66 (36.3%)	67 (36.0%)		142 (38.9%)

**(a):** 

Univariate Cox regression analysis		
Training set		
	HR (95% CI)	*p* value
Age	1.036 (1.008-1.065)	**0.012**∗
Gender	1.018 (0.585-1.771)	0.95
Grade		
Low grade	Reference	
High grade	22.430 (0.076-6621.401)	0.284
Stage		
Stage I+stage II	Reference	
Stage III+stage IV	4.818 (2.194-10.578)	**<0.001**∗
Tumor stage		
T0+T1+T2	Reference	**<0.001**∗
T3	3.202 (1.555-6.591)	0.002
T4	6.707 (2.914-15.438)	<0.001
Metastasis stage		
M0+Mx	Reference	
M1	2.428 (0.880-6.701)	0.087
Node stage		
N0+Nx	Reference	
N1+N2+N3	2.514 (1.546-4.090)	**<0.001**∗
Risk score group		
Low-risk group	Reference	
High-risk group	2.368 (1.425-3.935)	**0.001**∗
Testing set		
	HR (95% CI)	*p* value
Age	1.037 (1.013-1.061)	**0.002**∗
Gender	0.771 (0.475-1.252)	0.293
Grade		
Low grade	Reference	
High grade	21.095 (0.006-73263.326)	0.464
Stage		
Stage I+stage II	Reference	
Stage III+stage IV	2.358 (1.212-4.588)	**0.012**∗
Tumor stage		
T0+T1+T2	Reference	**0.044**∗
T3	1.993 (1.129-3.519)	0.017
T4	2.119 (1.017-4.417)	0.045
Metastasis stage		
M0+Mx	Reference	
M1	2.033 (0.281-14.705)	0.482
Node stage		
N0+Nx	Reference	
N1+N2+N3	2.410 (1.528-3.803)	<0.001∗
Risk score group		
Low-risk group	Reference	
High-risk group	1.845 (1.157-2.942)	0.010∗
Total set		
	HR (95% CI)	*p* value
Age	1.035 (1.017-1.054)	**<0.001**∗
Gender	0.884 (0.614-1.271)	0.505
Grade		
Low grade	Reference	
High grade	21.730 (0.229-2065.53)	0.185
Stage		
Stage I+stage II	Reference	
Stage III+stage IV	3.286 (1.978-5.458)	**0.002**∗
Tumor stage		
T0+T1+T2	Reference	<0.001∗
T3	2.403 (1.540-3.748)	<0.001∗
T4	3.469 (2.018-5.962)	<0.001∗
Metastasis stage		
M0+Mx	Reference	
M1	2.213 (0.904-5.415)	0.082
Node stage		
N0+Nx	Reference	
N1+N2+N3	2.422 (1.741-3.370)	**<0.001**∗
Risk score group		
Low-risk group	Reference	
High-risk group	2.060 (1.463-2.900)	**<0.001**∗

**(b):** 

Multivariate Cox regression analysis		
Training set		
	HR (95% CI)	*p* value
Age	1.032 (1.001-1.064)	0.052
Stage		
Stage I+stage II	Reference	
Stage III+stage IV	10.176 (2.318-46.455)	
Tumor stage		
T0+T1+T2	Reference	**0.002**∗
T3	0.342 (0.087-1.342)	0.016∗
T4	0.768 (0.186-3.180)	
Node stage		
N0+Nx	Reference	0.124
N1+N2+N3	1.494 (0.883-2.529)	0.716
Risk score group		
Low-risk group	Reference	0.134
High-risk group	2.163 (1.280-3.656)	**0.004**∗
Testing set		
	HR (95% CI)	*p* value
Age	1.036 (1.012-1.060)	0003∗
Stage		
Stage I+stage II	Reference	
Stage III+stage IV	0.890 (0.277-2.855)	
Tumor stage		
T0+T1+T2	Reference	0.844
T3	1.744 (0.993-3.170)	0.084
T4	2.207 (1.022-4.767)	
Node stage		
N0+Nx	Reference	0.053
N1+N2+N3	1.865 (1.160-3.001)	0.044
Risk score group		
Low-risk group	Reference	**0.010**∗
High-risk group	1.642 (1.013-2.661)	**0.044**∗
Total set		
	HR (95% CI)	*p* value
Age	1.033 (1.014-1.050)	**<0.001**∗
Stage		
Stage I+stage II	Reference	
Stage III+stage IV	1.868 (0.793-4.405)	0.153
Tumor stage		**<0.001**∗
T0+T1+T2	Reference	
T3	1.871 (1.184-2.957)	0.007
T4	2.865 (1.624-5.052)	<0.001∗
Node stage		
N0+Nx	Reference	
N1+N2+N3	1.834 (1.297-2.592)	**0.001**∗
Risk score group		
Low-risk group	Reference	
High-risk group	1.848 (1.305-2.618)	**0.001**∗

^∗^Statistically significant to predict overall survival rate.

## Data Availability

Data used during the current study are available from the authors on reasonable request for noncommercial use.

## Supplementary Materials

Supplementary Materials Supplementary Figure 1: the different methylated genes in normal tissues and bladder cancer samples.
