# Supplementary material for: A Three Protein-Coding Gene Prognostic Model Predicts Overall Survival in Bladder Cancer Patients
Source: Biomed Res Int. 2020 Oct 10;2020:7272960. doi: 10.1155/2020/7272960 (PMC7603549; doi:10.1155/2020/7272960)
Supplement: Supplementary Materials — Supplementary Figure 1: the different methylated genes in normal tissues and bladder cancer samples. [file 7272960.f1.docx]

**A three protein-coding gene prognostic model predicts overall survival in bladder cancer patients**

**Authors**:

Xiang-hui Ning^1^, Yuan-yuan Qi^2^, Song-chao Li^1,^ Zhan-kui Jia^1^, Jin-jian Yang^1*^

**Affiliation**:

1. Department of Urology, the First Affiliated Hospital of Zhengzhou University

2. Department of Nephrology, the First Affiliated Hospital, Zhengzhou University

*** Correspondence**:

Jin-jian Yang is the corresponding author

Department of Urology, the First Affiliated Hospital of Zhengzhou University, No.1 Eastern Jianshe Road, Er Qi District, Zhengzhou, Henan 450052, P.R China.

E-mail address: [yangjinjian2011@163.com](mailto:yangjinjian2011@163.com)

**Key words:**

Bladder cancer, protein coding gene, methylation, prognosis.

**Supplementary Figure 1**


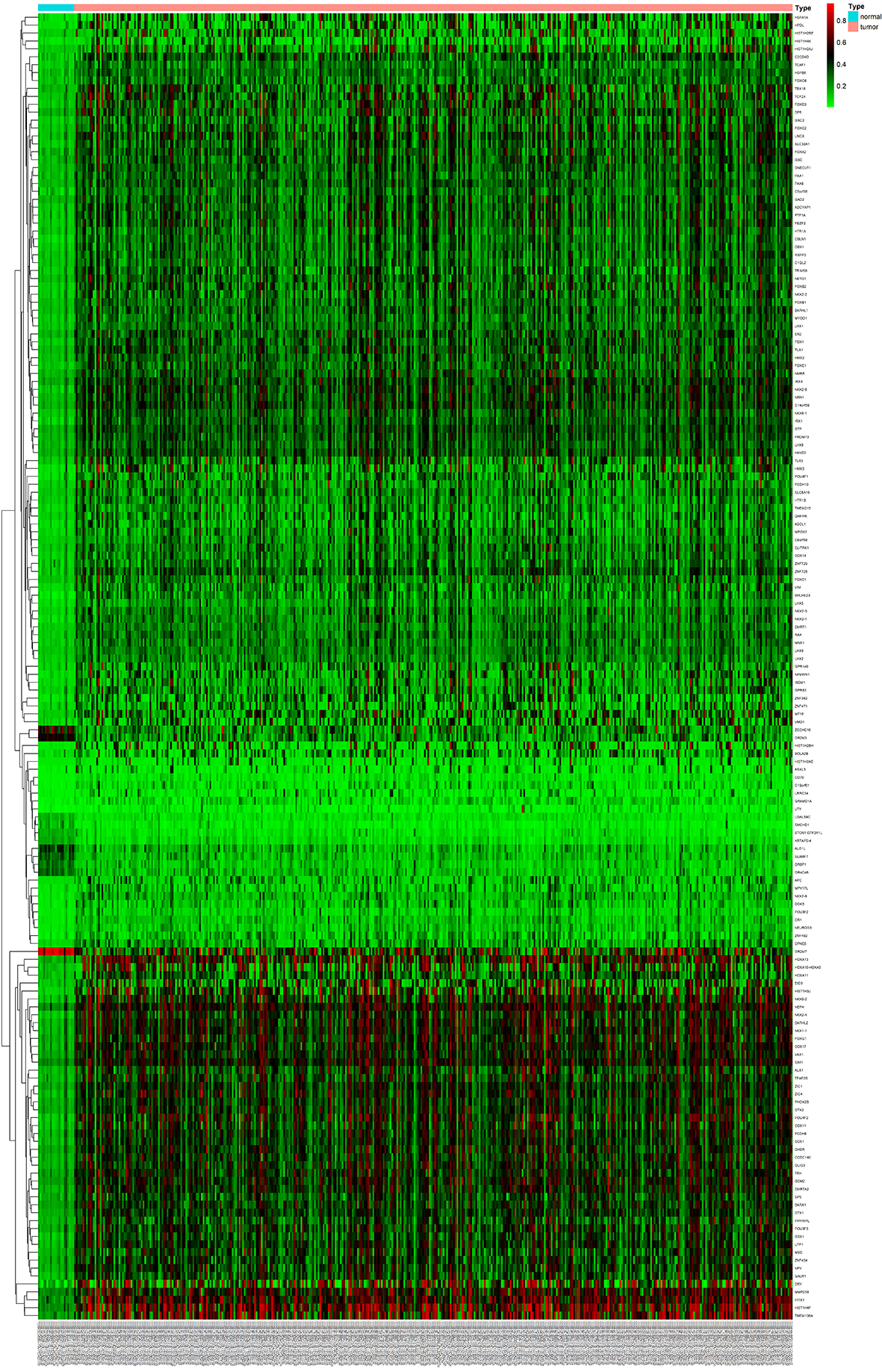


**Figure Legends**

**Figure 1.** The different methylated genes in normal tissues and bladder cancer samples.
